# In vivo antimalarial effect of 1-hydroxy-5,6,7-trimethoxyxanthone isolated from *Mammea siamensis* T. Anders. flowers: pharmacokinetic and acute toxicity studies

**DOI:** 10.1186/s12906-024-04427-z

**Published:** 2024-03-23

**Authors:** Prapaporn Chaniad, Arnon Chukaew, Prasit Na-ek, Gorawit Yusakul, Litavadee Chuaboon, Arisara Phuwajaroanpong, Walaiporn Plirat, Atthaphon Konyanee, Abdi Wira Septama, Chuchard Punsawad

**Affiliations:** 1 School of Medicine, Walailak University, Nakhon Si Thammarat, 80160 Thailand; 2 Research Center in Pathobiology and Tropical Medicine, Walailak University, Nakhon Si Thammarat, 80160 Thailand; 3 Chemistry Department, Faculty of Science and Technology, Suratthani Rajabhat University, Surat Tani, 84100 Thailand; 4 School of Pharmacy, Walailak University, Nakhon Si Thammarat, 80160 Thailand; 5 Research Center for Pharmaceutical Ingredient and Traditional Medicine, Cibinong Science Center, National Research and Innovation Agency (BRIN), West Java, 16915 Indonesia

**Keywords:** Antimalarial activity, *Mammea siamensis*, Pharmacokinetic, 1-Hydroxy-5,6,7-trimethoxyxanthone

## Abstract

**Background:**

The potent antiplasmodial activity of 1-hydroxy-5,6,7-trimethoxyxanthone (HTX), isolated from *Mammea siamensis* T. Anders. flowers, has previously been demonstrated in vitro. However, its in vivo activity has not been reported. Therefore, this study aimed to investigate the antimalarial activity and acute toxicity of HTX in a mouse model and to evaluate the pharmacokinetic profile of HTX following a single intraperitoneal administration.

**Methods:**

The in vivo antimalarial activity of HTX was evaluated using a 4-day suppressive test. Mice were intraperitoneally injected with *Plasmodium berghei* ANKA strain and given HTX daily for 4 days. To detect acute toxicity, mice received a single dose of HTX and were observed for 14 days. Additionally, the biochemical parameters of the liver and kidney functions as well as the histopathology of liver and kidney tissues were examined. HTX pharmacokinetics after intraperitoneal administration was also investigated in a mouse model. Liquid chromatography triple quadrupole mass spectrometry was used to quantify plasma HTX and calculate pharmacokinetic parameters with the PKSolver software.

**Results:**

HTX at 10 mg/kg body weight significantly suppressed parasitemia in malaria-infected mice by 74.26%. Mice treated with 3 mg/kg HTX showed 46.88% suppression, whereas mice treated with 1 mg/kg displayed 34.56% suppression. Additionally, no symptoms of acute toxicity were observed in the HTX-treated groups. There were no significant alterations in the biochemical parameters of the liver and kidney functions and no histological changes in liver or kidney tissues. Following intraperitoneal HTX administration, the pharmacokinetic profile exhibited a maximum concentration (C_max_) of 94.02 ng/mL, time to attain C_max_ (T_max_) of 0.5 h, mean resident time of 14.80 h, and elimination half-life of 13.88 h.

**Conclusions:**

HTX has in vivo antimalarial properties against *P. berghei* infection. Acute toxicity studies of HTX did not show behavioral changes or mortality. The median lethal dose was greater than 50 mg/kg body weight. Pharmacokinetic studies showed that HTX has a long elimination half-life; hence, shortening the duration of malaria treatment may be required to minimize toxicity.

## Background

Malaria is a major cause of morbidity and mortality throughout tropical and subtropical regions [1]. The disease is caused by protozoans from the genus *Plasmodium*. The most virulent parasite species responsible for severe clinical malaria and death is *Plasmodium falciparum* [2]. According to a World Health Organization report, in 2022, there were an estimated 247 million malaria cases and 625,000 deaths worldwide by 2020 [3]. Although artemisinin combined with other drugs, known as artemisinin-based combination therapies (ACTs), is the most effective way to treat malaria, reduced sensitivity, as evidenced by the slow clearance of *P. falciparum* parasites, has been reported. Artemisinin resistance was first reported in Cambodia in 2006 and has since spread to most of Southeast Asia. ACTs failures are increasing in the Greater Mekong Region, reaching 50%, which was observed in 140 patients when dihydroartemisinin-piperaquine was recently tested in 2015–2018 [4, 5]. *Plasmodium* resistance, especially *P. falciparum*, to currently available drugs poses a significant problem for treating and preventing malaria [6]. Increased resistance of malaria parasites to available drugs has encouraged researchers to continue searching for new and more effective antimalarials [7].

Medicinal plants are a potential source of new antimalarial drugs as they contain numerous compounds with diverse structures and pharmacological activities. These metabolites can be optimized to obtain better therapeutic agents [8]. Furthermore, medicinal plants have been used in traditional malaria treatment because of their efficacy, safety, low cost, and availability [9, 10]. Therefore, there is a need to identify new chemical compounds with antimalarial activity, especially from natural products, including plants and microorganisms, such as bacteria and fungi, which are rich sources of many bioactive substances [11]. The first antimalarial drug identified was quinine, which was originally isolated from the bark of the Cinchona species [12]. The current antimalarial drug of choice is artemisinin (Qinghaosu), which was initially derived from the leaves of *Artemisia annua* L [13].

*Mammea siamensis* T. Anders, locally known as Saraphi in Thailand, belongs to the Calophyllaceae family. Its flowers are traditionally used for treating heart problems and fever as well as to increase appetite [14]. A methanolic extract of the flowers of *M. siamensis* and its constituents have been reported to possess aromatase inhibitory activity [15], antiproliferative and apoptotic effects [16, 17], and the ability to suppress nitric oxide (NO) production [18]. In addition, our previous study found that the methanolic extract of the flowers of this plant exhibits antimalarial activity against the *Plasmodium falciparum* K1 strain with a half-maximum inhibitory concentration (IC_50_) value of 1.50 mg/mL [19]. In an in vitro study, 1-hydroxy-5,6,7-trimethoxyxanthone (HTX), its isolated compound, demonstrated strong antiplasmodial activity with an IC_50_ value of 9.57 µM [20]. This compound has not been tested for its efficacy in an in vivo model. Therefore, the present study aimed to investigate the antimalarial activity and acute toxicity of HTX in a mouse model and to evaluate the pharmacokinetic profile of this compound.

## Methods

### Plant materials

*M. siamensis* flowers were purchased from a Thai traditional drug store in Nakhon Si Thammarat Province, Thailand. The collection of plant materials followed the relevant guidelines and regulations of The Plant Varieties Protection, Department of Agriculture, Ministry of Agriculture and Cooperatives, Thailand. The plant was identified by Assoc. Prof. Tanomjit Supavita, a botanist at the School of Pharmacy at Walailak University. The plant material was deposited at the School of Medicine, Walailak University, with identification voucher number SMD122006002. Plant materials were rinsed with distilled water before being dried in a hot air oven at 60 °C and pulverized to coarse powder using a herb grinder.

### General experimental procedure

Silica gel (230–400 mesh, Sili Cycle Inc., Canada) was used for column chromatography (CC). All solvents were purchased from Labscan (Thailand) and were of analytical grade. All reagents were purchased from Sigma-Aldrich (USA). The nuclear magnetic resonance (NMR) spectra were obtained using an Avance NEO spectrometer (Bruker) at 500 MHz for ^1^H.

### Extraction and isolation of compounds

The dried *M. siamensis* flower powder (8.0 kg) was macerated extensively in dichloromethane (15 L) for 72 h with occasional shaking. The solvent was filtered through Whatman No. 1 filter paper. The residue was re-macerated for 72 h, and this procedure was repeated thrice. The filtrates were combined and concentrated using a rotary evaporator (Rotavapor®, Buchi, China) at 50 °C to obtain a brownish viscous crude extract (76.8 g). Finally, the dried crude extract was kept in screw-cap containers and stored at 4 °C for further use.

Based on its in vitro antiplasmodial activity, HTX was isolated according to our previous study, with some modifications [20]. The dichloromethane extract (75 g) underwent quick CC (QCC) through silica gel by step-gradient elution starting with dichloromethane. The polarity was increased with ethyl acetate (EtOAc) and acetone to yield 12 fractions (F1-F12), based on thin-layer chromatography profiles. Fraction 6 (8.2 g) was purified by QCC using 10% EtOAc in hexane to produce five subfractions (6A-6E). Subfraction 6B (4.13 g) was further purified by QCC using 15% EtOAc in hexane to obtain four subfractions (6B1-6B4). Subfraction 6B2 (2.66 g) was further purified by CC with 15% EtOAc in hexane to obtain compound **1** as a yellow powder (62.6 mg).

### Animals

Healthy adult male Institute of Cancer Research (ICR) mice weighing 25–30 g body weight and aged 6–8 weeks were obtained from Nomura Siam International Co., Ltd., Pathumwan, Bangkok, Thailand, for antimalarial activity, acute toxicity, and pharmacokinetic testing. The study was approved by the Animal Ethics Committee of Walailak University (certificate number: WU-ACUC-65015), and in accordance with Ethical Principles and Guidelines for the Use of Animals by the National Research Council of Thailand. All mice were housed in cages under standard environmental conditions at a relative humidity of 50–60% and a room temperature of about 22 °C (± 3 °C) under a natural 12-hour dark-light cycle. Mice had free access to pellet diet and clean drinking water. All mice were acclimatized to the experimental environment for a week before beginning the experiment. This study was reported in accordance with ARRIVE guidelines.

### Parasite inoculation

Wild-type *P. berghei* ANKA strain was obtained from Thomas F. McCutchan (BEI Resources, NIAID, NIH). *P. berghei*-infected red blood cells were administered intraperitoneally to donor mice. When parasitemia levels reached 20–30%, blood was collected from the heart using a cardiac puncture procedure and placed in a vacutainer heparinized tube for injection into experimental animals [21].

### In vivo 4-day suppressive test of *P. berghei*

A 4-day suppressive test was used to measure the schizonticidal activity of HTX in *P. berghei*-infected ICR mice. This test was performed according to a previous method, with some modifications [22, 23]. Male ICR mice were randomly divided into six groups, each consisting of five mice, as shown in Table 1. All mice were intraperitoneally injected with 1 × 10^7^*P. berghei* ANKA-parasitized erythrocytes. Treatment was initiated four hours after the mice were infected with *Plasmodium* parasite on the first day (D0). The negative control group was administered 150 µl of 2% dimethyl sulfoxide (DMSO) in normal saline solution (NSS), whereas the positive control group was administered 3 mg/kg body weight of artesunate and chloroquine intraperitoneally each day. For each experimental group, animals received daily intraperitoneal doses of 1, 3, or 10 mg/kg body weight of HTX. Mice received each substance daily for 4 days (D_0_-D_3_). On the fifth day (D_4_), parasitemia was determined using Giemsa-stained thin blood smears prepared from the tails of each mouse. Mice were euthanized at the end of the experiment by intraperitoneal injection of pentobarbital (200 mg/kg body weight).

Finally, the percentage suppression of parasite growth was calculated using the following equation:

$$\% {\text{inhibition = }}\frac{\begin{gathered}{\text{(parasitemia of negative group - }} \hfill \\{\text{ parasitemia of treated group)}} \hfill \\ \end{gathered} }{{{\text{(parasitemia of negative group)}}}}{\text{ }} \times {\text{ 100}}$$

**Table 1:** Group classifications and doses used in the 4-day suppressive test

Groups (*n* = 5/group)	Agent	Dose (mg/kg)
Negative control	2% DMSO in NSS	-
Positive control 1	Artesunate	3
Positive control 2	Chloroquine	3
Experimental group 1	HTX	1
Experimental group 2	HTX	3
Experimental group 3	HTX	10

Abbreviations: HTX, 1-hydroxy-5,6,7-trimethoxyxanthone; NSS, normal saline solution.

### Acute toxicity test

Acute HTX toxicity was determined in non-infected ICR mice aged 6–8 weeks and weighing 25–35 g using the Organization for Economic Cooperation and Development standard criteria [24]. Fifteen mice were randomly divided into three groups (*n* = 5): untreated, negative control, and HTX-treated. All mice fasted for three hours prior to the experiment, and only drinking water was available. HTX was dissolved in 2% DMSO in NSS at 50 mg/kg body weight. In the treatment group, the first mouse was intraperitoneally given a single dose of 50 mg/kg body weight HTX, whereas in the negative control group, the first mouse was intraperitoneally given 200 µL of 2% DMSO in NSS. After dosing, mice were observed for 24 h for gross physiological and behavioral changes, such as sleep, hair erection, poor appetite, convulsions, diarrhea, lacrimation, salivation, mortality, and other manifestations of toxicity. If death was not observed within 24 h in the first mouse, the same dose was administered to 4 more mice, and they were observed for signs and symptoms of toxicity for 14 days. All mice were anaesthetized using pentobarbital (60 mg/kg body weight) by intraperitoneal injection. Blood samples were collected via cardiac puncture into heparinized tubes for biochemical analyses. The liver and kidney tissues were collected for histological examination. The mice were subsequently euthanized with an intraperitoneal injection of pentobarbital (200 mg/kg body weight).

### Histopathological examination

Histopathological examinations of liver and kidney tissues were performed in accordance with standard histological protocols, as reported previously [25, 26]. In brief, all tissues were fixed in 10% buffered formalin, dehydrated using a gradient series of ethanol solutions, cleaned with xylene, and deposited in a paraffin mold. The paraffin blocks were then sectioned to a thickness of 5 μm using a rotary microtome, transferred to glass slides, and stained with hematoxylin and eosin (H&E) solution. The stained slides were examined under light microscopy by two independent observers who were blinded to the experimental groups for examining the histological changes.

### In vivo pharmacokinetic study

#### Samples preparation

A pharmacokinetic study was conducted in non-infected ICR mice aged 6–8 weeks and weighing 25–35 g. Male ICR mice were randomly divided into two groups, each consisting of five mice. The first group is a negative control group and was injected with 150 µL of 2% DMSO in NSS, whereas the second group is a test group that was administered 5 mg/kg body weight of HTX compound intraperitoneally. Blood (50 L) was taken from the tail vein prior to administration of test compounds (0 h) and after at, 0.5, 1, 2, 3, 5, 8, 24, 48, and 72 h. Blood samples were collected in microtubes containing ethylenediaminetetraacetic acid (EDTA) and kept on ice until they were centrifuged at 3,000 × g for 10 min at 4 °C to allow the collection of plasma, which was stored at − 80 °C.

#### Liquid chromatography triple quadrupole mass spectrometry (LC-MS/MS)

LC-MS/MS (Agilent 1290 infinity LC and Agilent 6490 triple quadrupole mass spectrometer) with an electrospray ionization (ESI, Agilent Technologies, USA) source was used to detect HTX in the plasma. Using a VertiSepTM USP C18 column (4.6 mm × 150 mm, particle size = 5 μm; Vertical Chromatography Co., ltd., Nonthaburi, Thailand), the separation was achieved. Acetonitrile:1 mM formic acid (7:3) was used as the mobile phase and was maintained at a constant flow rate of 0.5 mL/min. The injection volume was 1 µL, and column temperature was maintained at 30 °C. The following operating conditions for the MS were optimized: the source temperature was kept at 200 °C, the ion spray voltage for the positive mode was set at 3 KV, and the collision energy for HTX was set at 43 V. Nitrogen was used as the collision gas. The nebulizer and sheath gas flow rates were set to 14 and 11 L/min, respectively. The MassHunter qualitative analysis software was used to acquire the data (Agilent Technologies, Inc. Headquarters, CA, USA). HTX was quantified using the transitions for HTX at m/z 303→199 in the multiple reaction monitoring (MRM) mode.

#### Preparation of the standard and quality control samples

By dissolving precisely weighed HTX and reference compounds in acetonitrile, stock HTX solutions were prepared. By diluting the stock solutions using a mixture of acetonitrile and water (2:8, v/v), a series of working solutions with concentrations between 7.81 and 1000 ng/mL were prepared. All solutions were stored at 4 °C. By spiking the blank plasma (50 µL) with 25 µL of standard working solutions, HTX calibration standards (0.78, 1.56, 3.13, 6.25, 12.5, 25.0, 50, 125, and 250 ng/mL) were prepared. The extraction solvent ethyl acetate (0.5 mL) was added to 75 µL of HTX-spiked plasma. The sample was vortexed for 1 min, followed by ultrasonic vibration for 15 min. Then, centrifugation for 10 min at 15,000 × g was done. The upper organic layer was transferred to a new microtube and dried via evaporation. The dried extract was reconstituted in 150 µL of 50% (v/v) acetonitrile in water. HTX recovery experiments were performed to confirm extraction efficacy. The effectiveness of liquid-liquid extraction was then determined by comparing the peak regions of the extracted HTX-spiked plasma with those of HTX spiked with blank plasma extract.

#### Pharmacokinetic analysis of HTX

The plasma concentration-time profiles of HTX were determined by non-compartmental analysis with PKSolver 2.0 software. Pharmacokinetic parameters were calculated to estimate the areas under the concentration-time curve (AUCs) from 0 to 48 h and from 0 to ∞, elimination half-life (t_1/2_), maximum concentration achieved (C_max_), time to attain C_max_ (T_max_), mean residence time (MRT), apparent volume of distribution, and clearance. Additionally, the apparent volume of the central or plasma compartment, apparent volume of the peripheral compartment, transfer rate constant from the central compartment to the peripheral compartment, and transfer rate constant from the peripheral to central compartment were calculated using the two-compartment model.

### Statistical analysis

Results were presented as means ± SEM. IBM SPSS Statistics version 23.0 software (SPSS, IL, USA) was used for statistical analysis. Normal distribution was tested using the Kolmogorov–Smirnov goodness-of-fit test. Statistical significance of parasitemia inhibition was determined using one-way ANOVA, followed by Tukey’s multiple comparison test. Statistical significance was set at 0.05 (*p* < 0.05).

## Results

### Identification of compound 1

**Fig. 1:**
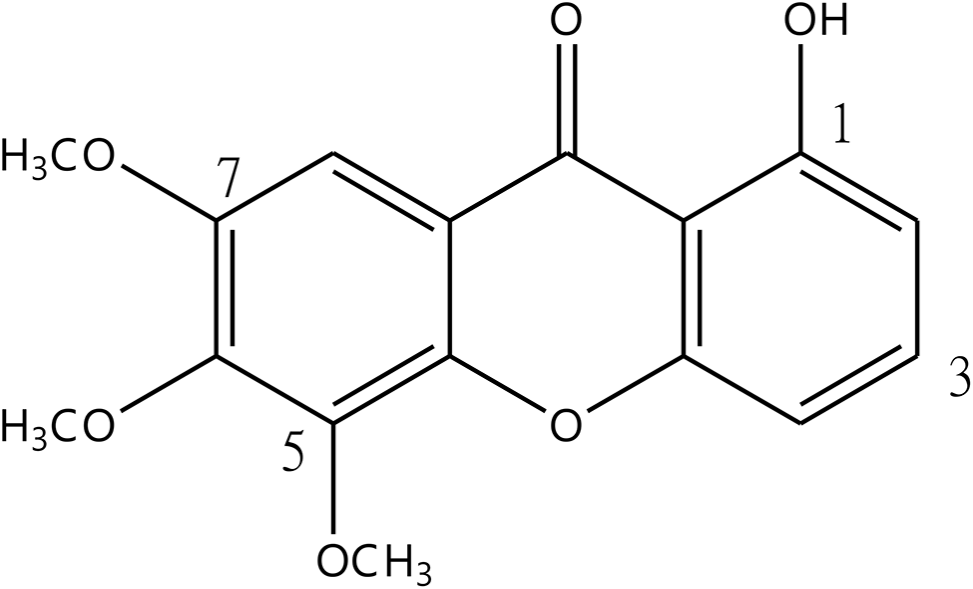
Structure of HTX compound

The structures of compound **1 (**Fig. 1**)** was elucidated by ^1^H-NMR analysis and confirmed by comparison with previously reported data [27]. Compound **1-hydroxy-5,6,7-trimethoxyxanthone (1)**: Yellow powder. ^1^H-NMR (acetone-*d*_6_, 500 MHz): *δ* 6.93 (1H, d, *J* = 8.3 Hz, H-2), 7.13 (1H, d, *J* = 8.3 Hz, H-4), 7.19 (1H, s, H-8), 7.68 (1H, t, *J* = 8.3 Hz, H-3), 12.68 (1H, brs, 1-OH), 3.94 (3H, s, 5-OCH_3_), 3.93 (3H, s, 6-OCH_3_), 3.95 (3H, s, 7-OCH_3_).

### Antimalarial activity

The antimalarial effect of HTX was examined in a 4-day suppressive test as a standard model. Mice in each group received daily intraperitoneal doses of HTX at different concentrations (1, 3, and 10 mg/kg) body weight. The percentages of parasitemia and suppression are presented in Table 2. The results showed that HTX effectively suppressed *Plasmodium* parasites and showed significant (*p* < 0.05) antimalarial activity against *P. berghei* infection in mice compared to that of negative controls. The parasite counts decreased in a dose-dependent manner after HTX treatment. The highest level of suppression (74.26%) was obtained with a 10 mg/kg dose of HTX, followed by a dose of 3 mg/kg (46.88%), whereas the lowest level of suppression (34.56%) was obtained with a dose of 1 mg/kg. However, antimalarial activity against *P. berghei* infection at all doses was significantly (*p* < 0.05) lower than that of the positive control groups with standard drugs, artesunate and chloroquine (3 mg/kg each), which showed 91.26% and 92.07% suppression, respectively.

**Table 2:** Effects of HTX against *Plasmodium* parasites in malaria-infected mice

Groups	Dose (mg/kg)	% parasitemia	% suppression
Negative control	-	33.49 ± 3.01	-
Artesunate	3	3.84 ± 0.54	91.26 ± 3.54 ^c, d, e^
Chloroquine	3	3.42 ± 0.35	92.07 ± 4.21 ^c, d, e^
HTX	1	21.91 ± 2.25	34.56 ± 4.91 ^a, b, d, e^
HTX	3	17.79 ± 2.31	46.88 ± 3.96 ^a, b, c, e^
HTX	10	8.62 ± 1.21	74.26 ± 3.32 ^a, b, c, d^

Data represent the mean ± SEM (*n* = 5 per group). Values are significantly different at *p* < 0.05 : different superscript letters. ^a^compared with artesunate; ^b^compared with chloroquine; ^c^compared with 1 mg/kg HTX; ^d^compared with 3 mg/kg HTX; ^e^compared with 10 mg/kg HTX.

### Acute toxicity

Acute toxicity study at 50 mg/kg HTX demonstrated no visible signs of physical and behavioral changes, such as sleep, hair erection, poor appetite, convulsions, diarrhea, lacrimation, salivation, mortality, and other manifestations of toxicity during the experimental period. No mortality was observed in any of the mice within 24 h of HTX administration or the following 14 days. Therefore, the lethal dose of HTX appears greater than 50 mg/kg body weight.

### Effects of HTX on the liver and kidney functions

The effects of 50 mg/kg HTX on the biochemical parameters of the liver and kidneys are shown in Table 3. The biochemical parameters of liver function, including aspartate aminotransferase (AST), alanine aminotransferase (ALT), and alkaline phosphatase (ALP), in the HTX-treated group were 46.20, 13.00, and 105.80 U/L. These were not significantly different from those in the untreated and negative controls (*p* > 0.05). Additionally, kidney function was evaluated based on plasma blood urea nitrogen (BUN) and creatinine levels, which were 16.00 and 0.25 mg/dL, respectively, in mice treated with 50 mg/kg HTX. These values were not significantly different compared to those of the untreated control (*p* > 0.05) after 14 days of exposure to HTX. These results showed that HTX did not affect the liver and kidney functions.

**Table 3:** Effects of HTX on the biochemical parameters of the liver and kidney functions in the acute toxicity test

Parameters	Untreated control	Negative control	HTX
Liver function test			
AST (U/L)	44.00 ± 0.55	44.20 ± 2.27	46.20 ± 2.35
ALT (U/L)	14.60 ± 1.29	15.60 ± 1.15	13.00 ± 3.63
ALP (U/L)	108.60 ± 3.86	106.20 ± 2.27	105.80 ± 2.92
Kidney function test			
BUN (mg/dL)	16.80 ± 0.58	18.20 ± 0.33^c^	16.00 ± 0.55^b^
Creatinine (mg/dL)	0.26 ± 0.01	0.27 ± 0.01	0.25 ± 0.02

Data represent the mean ± SEM (*n* = 5 per group). Values are significantly different at *p* < 0.05 : different superscript letters; ^a^Compared with untreated control; ^b^compared with negative control (2% DMSO in NSS); ^c^compared with 50 mg/kg HTX.

### Histopathological examination of the livers and kidneys of the mice

**Fig. 2:**
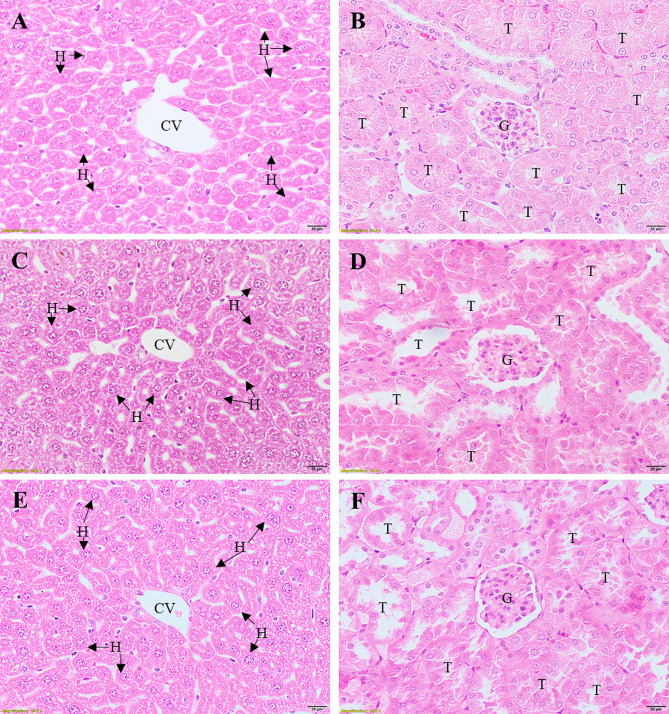
Histopathological examination of the liver and kidney sections of mice. Mice were intraperitoneally administered with 2% DMSO in normal saline solution (negative control) or HTX 50 mg/kg. Liver and kidney were excised and stained with hematoxylin and eosin. Untreated control group (**A**, **B**), negative control group (**C**, **D**), 50 mg/kg HTX-treated group (**E**, **F**). All images are 400 × magnification. Bars = 20 μm. T: tubules; G: glomerulus; CV: central vein; H: hepatocyte. Abbreviation: HTX, 1-hydroxy-5,6,7-trimethoxyxanthone

The liver and kidney sections from mice were analyzed histologically using H&E staining. Typical histological structures in different parts of the liver and kidneys of mice treated with 50 mg/kg HTX are shown in Fig. 2. The results demonstrated normal histology in the livers of mice treated with HTX, including the morphological characteristics of the hepatocytes, such as arrangement, size, and color staining. Moreover, inflammatory cell infiltration and sinusoidal vasodilation were not detected (Fig. 2E) when compared with the untreated control (Fig. 2A) and negative control groups (Fig. 2C). Histopathological changes in the kidneys were also investigated. The histological features of the kidneys in the HTX-treated group were also normal, including kidney epithelial cells, glomeruli, and Bowman’s capsules (Fig. 2F), compared with those in the untreated control (Fig. 2B) and negative control groups (Fig. 2D).

### Mass spectrum

**Fig. 3:**
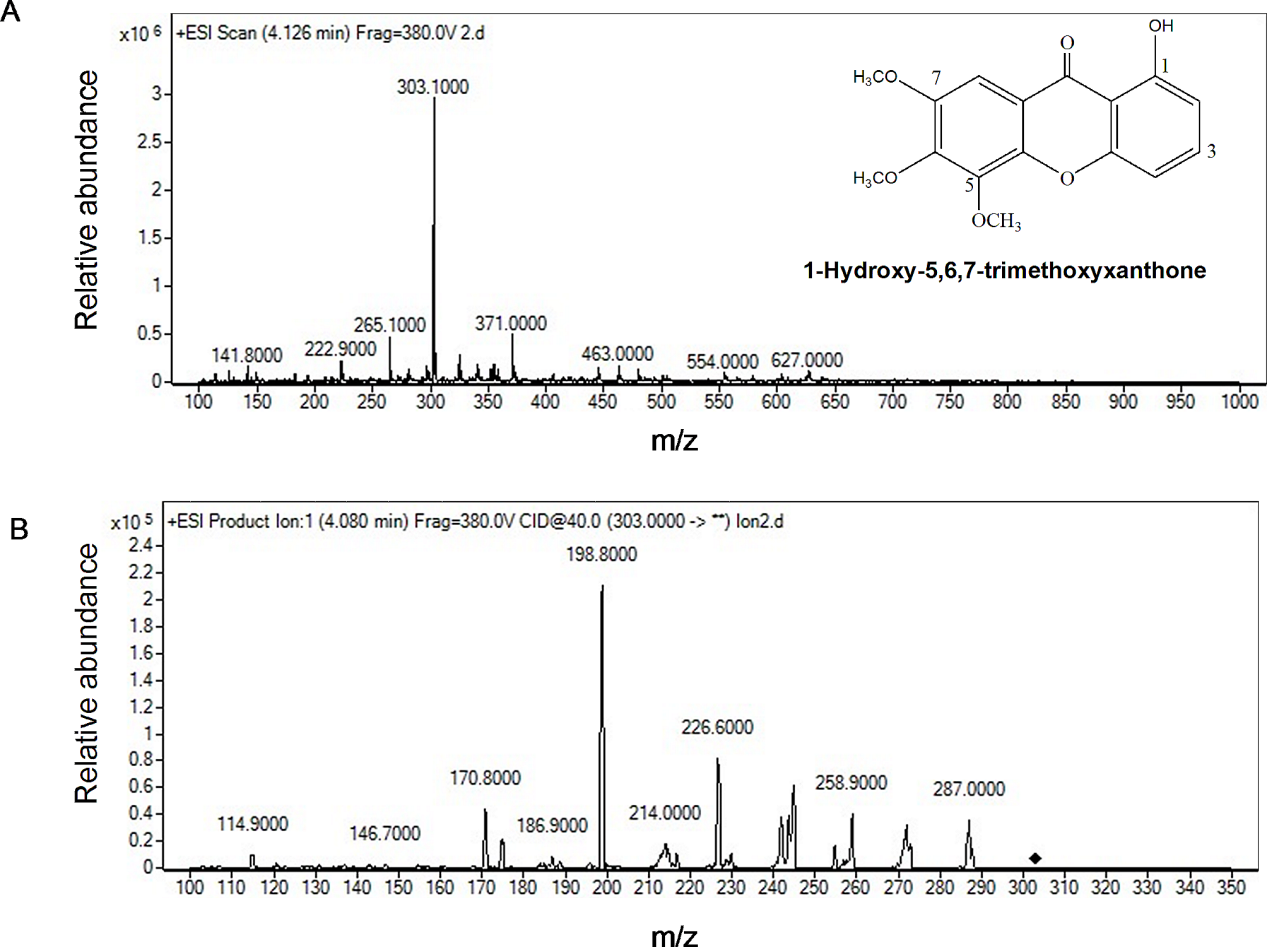
The molecular weight of HTX ([M-H]^+^, **A)** and the product ion of HTX **(B)**. Abbreviation: HTX, 1-hydroxy-5,6,7-trimethoxyxanthone

The mass spectrum of HTX [M + H]^+^ showed a peak at m/z 303 (Fig. 3A). The product ion with the highest relative abundance when the collision energy was optimized was at 198.8 m/z (Fig. 3B). The findings showed that the 43 V collision energy generated most of the aforementioned ion product, which was subsequently subjected to MRM mode analysis. HTX had a retention period of 4.126 min.

### Calibration, linearity, precision, and recovery

The calibration curve was linear for HTX concentrations in mouse plasma, ranging from 0.781 to 250 ng/mL, with a correlation value of R^2^ = 0.9978 (y = 1527x–1332). The precision of analysis in the concentration range was between 0.33 and 5.77% expressed as coefficient of variation. The signal-to-noise ratio at 0.781 ng/mL was 48.3 ± 8.1. Thus, a calibration curve was established with an HTX concentration range of 0.781–250 ng/mL. The LC-MS/MS system specifically detected HTX in plasma samples. The efficacy of the liquid-liquid extraction was confirmed (Table 4). The recovery was in the range of 87.2–106.3%; thus, the extraction method is efficient for recovering HTX from the sample.

**Table 4:** Recovery of HTX by liquid-liquid extraction in plasma

Concentration (ng/mL)	% Recovery of HTX (*n* = 3)
250	87.2 ± 0.2
25.0	106.3 ± 5.0
0.781	101.1 ± 7.9

### Pharmacokinetic analysis of HTX

**Fig. 4:**
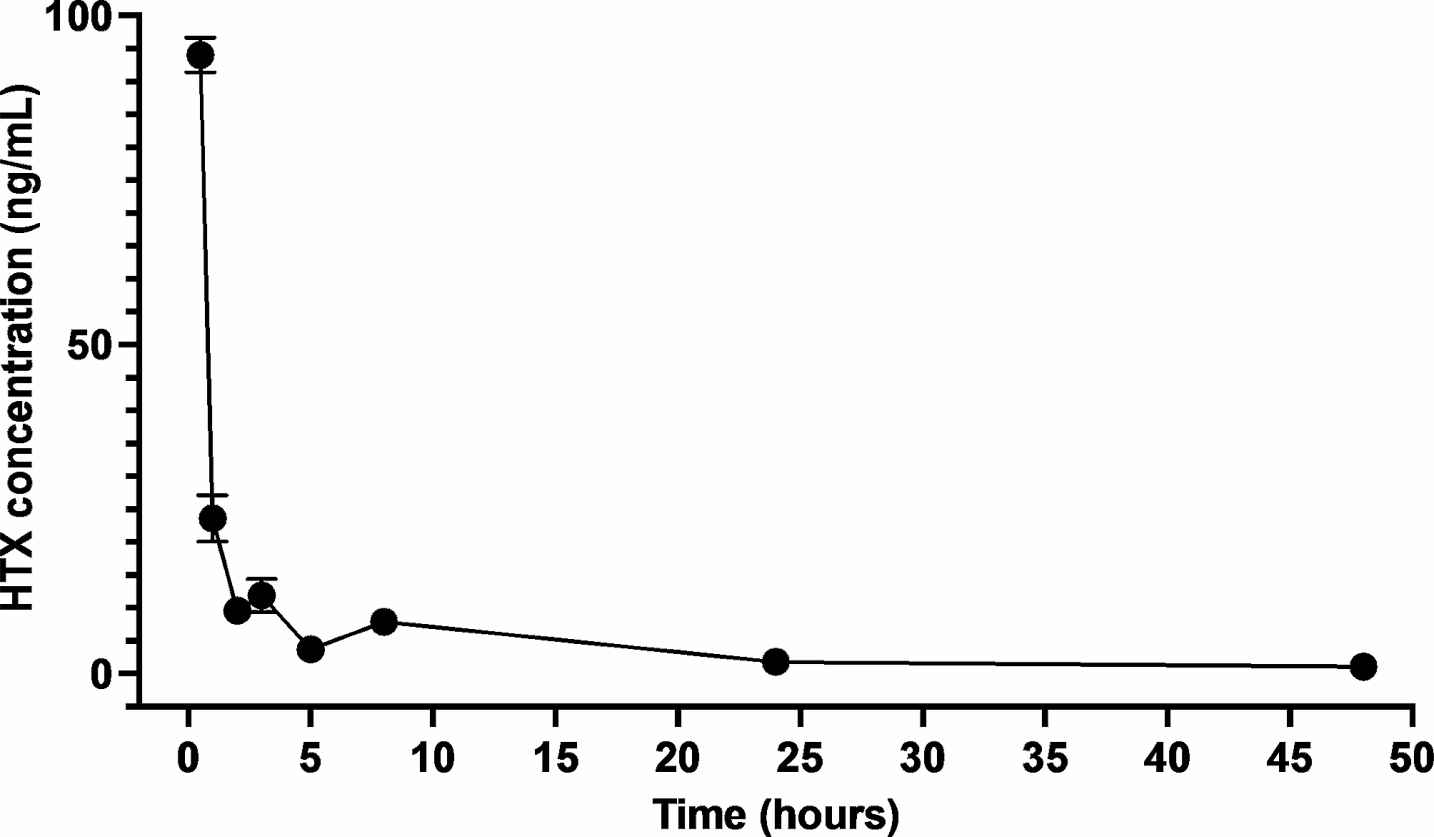
Plasma concentration-time curve of HTX following intraperitoneal administration at 5 mg/kg dose in ICR mice. Data expressed as mean ± standard deviation from five rats at different time points

Pharmacokinetic parameters of HTX are presented in Table 5. These results showed that HTX exhibited a C_max_ of 94.02 ng/mL, T_max_ of 0.5 h, MRT of 14.79 h, and a t_1/2_ of 13.88 h. The plasma concentration versus time profiles of HTX after intraperitoneal administration are illustrated in Fig. 4.

**Table 5:** Pharmacokinetic parameters of HTX

Parameter	Value
AUC_0 − 48 h_ (ng∙h/mL)	226.200 ± 4.742
AUC_0−∞_ (ng∙h/mL)	247.227 ± 5.325
T_max_ (h)	0.500 ± 0.021
C_max_ (ng/mL)	94.020 ± 3.214
t_1/2_ (h)	13.881 ± 1.231
MRT_0−∞_ (h)	14.795 ± 0.515
CL/F (mg/kg)/(ng/mL)/h	0.020 ± 0.002
Vz/F (mg/kg)/(ng/mL)	0.405 ± 0.014
V_1_ (mg/kg)/(ng/mL)	1.633 ± 0.003
V_2_ (mg/kg)/(ng/mL)	19.808 ± 2.031
K_12_ (1/h)	1.888 ± 0.013
K_21_ (1/h)	0.156 ± 0.002

Data expressed as mean ± standard deviation (*n* = 5). Abbreviations: AUC_0 − 48_: area under the curve from 0 to 48 h; AUC_0−∞_: area under the curve from 0 to infinity; t_max_: time to reach maximum concentration; C_max_: maximum concentration; t_1/2_: elimination half-life; MRT_0−∞_: mean residence time from time zero to infinity; CL/F: apparent total clearance of the drug; Vz/F: apparent volume of distribution during terminal phase after non-intravenous administration; V1: apparent volume, central or plasma compartment in a two-compartment model; V2: apparent volume, peripheral compartment in a two-compartment model; K12: rate constant, central to peripheral compartment; K21: rate constant, peripheral to central compartment.

## Discussion

The present study investigated the antimalarial activity of HTX against *P. berghei* infection in mice as well as the acute toxicity and pharmacokinetic parameters. Here, the in vivo antimalarial activity of HTX was first investigated. Based on our previous study of the antimalarial properties of several compounds isolated from *Mammea siamensis* flowers, HTX was shown to exhibit the most potent antimalarial effect against the *P. falciparum* K1 strain. This is a chloroquine-resistant strain, with an IC_50_ value of 9.57 µM [20]. The antimalarial effects of the xanthone (HTX), showed a dose-dependent pattern. A high dose of HTX (10 mg/kg body weight) exhibited a 74.26% decrease in *P. berghei*.

Xanthones, secondary metabolites from natural sources, contain various substituents on two benzene rings, thus leading to their rich structural diversity. They possess a broad spectrum of pharmacological properties, such as anticancer, antitumor, antioxidant, antidiabetic, and anticarcinogenic activities [28–32]. Xanthones also exhibit antimalarial activity. Α-Mangostin, a xanthone from *Garcinia mangostana*, is active against the resistant *P. falciparum* chloroquine-resistant (FCR3) strain with an IC_50_ value of 0.2 µM [33]. A previous study reported that xanthones exhibit antimalarial effects by forming complexes with heme and inhibiting hemozoin formation [34]. Therefore, the antimalarial activity of HTX may have resulted from inhibition of hemozoin formation, which has the same mechanism of action as chloroquine, an antimalarial drug [35, 36].

Because in vivo toxicology data for HTX have never been reported, the acute intraperitoneal toxicity of HTX in ICR mice was investigated. This study was performed using 50 mg/kg HTX, and toxicity symptoms were monitored for 14 days. The results showed that until the end of the experiment, all treated mice were alive without any symptoms of hair erection, convulsions, diarrhea, lacrimation, and salivation or other manifestations of toxicity. Additionally, biochemical analyses were performed to assess the liver and kidney functions. Since the liver is the major target organ of toxicity, liver damage may compromise the integrity of hepatocytes, causing the release of membrane-bound enzymes, such as ALT and AST. Damage to the hepatobiliary system results in the release of essential enzymes, such as ALP, and/or reduces the biosynthetic and catabolic capacities of the liver. High levels of liver enzymes are signs of hepatocellular toxicity, whereas a decrease may indicate an enzyme inhibition [37, 38]. However, IP administration is less likely to be toxic to the liver than oral administration because the liver’s first-pass effect is minimized ​ [39].

In the present study, intraperitoneal administration of HTX at 50 mg/kg body weight had no effect on AST, ALT, or ALP enzyme levels. The levels of these enzymes did not significantly differ between the treated and control groups. As the primary excretory organ, the kidney is a major target of drug-induced toxicity because it is naturally exposed to a greater proportion of circulating drugs and chemicals. As BUN and creatinine are excreted primarily by the kidneys, blood chemistry analyses that measure BUN and creatinine concentrations are most commonly used to evaluate renal function. High levels of BUN and creatinine have been detected in the blood [40, 41]. The present study revealed that an acute single dose of HTX did not alter blood BUN and creatinine levels compared to the control. The liver and kidney histology were also investigated to confirm the acute toxicity of HTX. The result showed that the HTX-treated group possessed normal hepatocyte histology, including morphology, cell size, arrangement, and color staining. For kidney histopathology, HTX did not present with lesions and had normal glomeruli, Bowman’s capsules, and renal cells.

Regarding acute toxicity, biochemical markers of the liver and kidney, consistent with the results of the histopathological examination, showed that HTX did not produce any abnormalities and did not cause mortality up to a dose of 50 mg/kg body weight. These results suggest that intraperitoneal (IP) administration of HTX is relatively safe and has a median lethal dose (LD_50_) greater than 50 mg/kg body weight. Generally, if the LD_50_ of a test substance is three times greater than the minimum effective dose (MED), it is considered a good candidate for further studies [42]. However, HTX showed no lethality in mice at 50 mg/kg, which is more than three times the MED. Gross physical and behavioral observations of experimental mice revealed no visible signs of acute toxicity, indicating that HTX is safe.

Pharmacokinetic parameters aid in understanding the body’s reactions to drugs and have several uses in toxicology and biopharmaceutics [43]. The study of pharmacokinetics of compounds for malaria treatment drug development is an important step toward further evaluation in clinical trials [44]. In this study, we selected the IP route because it is easy, quick, minimally stressful for animals and suitable for our test compound, which is poorly soluble and avoids gastrointestinal tract and potential degradation of the compound [45]. The concentration-time curve of HTX showed fluctuations that may be attributed to the pharmacokinetic phenomenon of tissue distribution [43]. The calculated elimination t_1/2_ after the IP administration of HTX was 13.88 h. This represents a relatively long elimination half-life when compared to those of other antimalarial drugs, such as artesunate (0.8–1.2 h) [46], dihydroartemisinin (0.9–2 h [47, 48], and arterolane (2–4 h) [49]. Drugs with an elimination half-life longer than that of current antimalarial drugs may shorten the duration of malaria treatment. This would reduce the cost of current therapies [50].

Regarding malaria treatment, WHO recommends using triple artemisinin-based combination therapy (TACT), which combines short-acting artemisinin and two long-acting partner drugs [3]. The short but rapidly acting artemisinin quickly reduces the total number of parasites circulating in a patient, thus reducing the stochiastic chances for the selection of resistant mutants in the remaining low number of parasites. A longer-acting partner drug is continued suppression, complete clearance of parasites, and prevention of drug resistance [51].

## Conclusions

This is the first report on the antimalarial activity of HTX in a mouse model. This study demonstrated that HTX possessed potent antimalarial activity against *P. berghei*. A single IP injection of HTX resulted in no behavioral changes or mortality. The LD_50_ of HTX was greater than 50 mg/kg body weight. Pharmacokinetics study showed that HTX has a long elimination half-life of 13.88 h. Further studies are required to elucidate the mechanism of action of HTX against malarial infections. Furthermore, this compound should be tested in combination with other antimalarial drugs to better assess its efficacy and adverse effects. This study can be considered an important step in identifying new antimalarial lead compounds to address the emergence of antimalarial drug resistance.

## Data Availability

All data generated or analyzed during this study are included in this published article. Additional files are available from the corresponding author on reasonable request.

## Abbreviations

ACT: Artemisinin-based combination therapy
ALP: Alkaline phosphatase
ALT: Alanine aminotransferase
AST: Aspartate aminotransferase
AUC: Area under the concentration-time curve
CC: Column chromatography
C_max_: Maximum concentration achieved
H E: Hematoxylin and eosin
HTX: 1-Hydroxy-5,6,7-trimethoxyxanthone
ICR: Institute of Cancer Research
IP: Intraperitoneal
LC-MS/MS: Liquid chromatography triple quadrupole mass spectrometry
LD_50_: Median lethal dose
MED: Minimum effective dose
MRM: Multiple reaction monitoring
MRT: Mean residence time
NMR: Nuclear magnetic resonance
NSS: Normal saline solution
QCC: Quick column chromatography
t_1/2_: Elimination half-life
T_max_: Time to attain maximum concentration
